# Academic leaders must support inclusive scientific communities during COVID-19

**DOI:** 10.1038/s41559-020-1233-3

**Published:** 2020-06-03

**Authors:** Bea Maas, Kathleen E. Grogan, Yolanda Chirango, Nyeema Harris, Luisa Fernanda Liévano-Latorre, Krista L. McGuire, Alexandria C. Moore, Carolina Ocampo-Ariza, Monica Marie Palta, Ivette Perfecto, Richard B. Primack, Kirsten Rowell, Lilian Sales, Rejane Santos-Silva, Rafaela Aparecida Silva, Eleanor J. Sterling, Raísa R. S. Vieira, Carina Wyborn, Anne Toomey

**Affiliations:** 1 Department of Botany and Biodiversity Research, University of Vienna, Vienna, Austria; 2 Institute of Zoology, University of Natural Resources and Life Sciences, Vienna, Austria; 3 Departments of Anthropology & Biology, Pennsylvania State University, University Park, PA USA; 4 Department of Botany and Zoology, Stellenbosch University, Stellenbosch, South Africa; 5 Department of Ecology and Evolutionary Biology, University of Michigan, Ann Arbor, MI USA; 6 Conservation Biogeography Lab, Universidade Federal de Goiás, Goiânia, Brazil; 7 Programa de Pós-Graduação em Ecologia e Evolução, Universidade Federal de Goiás, Goiânia, Brazil; 8 Department of Biology, Institute of Ecology and Evolution, University of Oregon, Eugene, OR USA; 9 Center for Biodiversity and Conservation, American Museum of Natural History, New York, NY USA; 10 Agroecology, Georg-August University of Goettingen, Goettingen, Germany; 11 Department of Environmental Studies and Science, Pace University, New York, NY USA; 12 School for Environment and Sustainability, University of Michigan, Ann Arbor, MI USA; 13 Biology Department, Boston University, Boston, MA USA; 14 Environmental Studies Program, Regent Administrative Center, University of Colorado Boulder, Boulder, CO USA; 15 Department of Animal Biology, Institute of Biology, University of Campinas, Campinas, Brazil; 16 International Institute for Sustainability, Rio de Janeiro, Brazil; 17 W.A. College of Forestry and Conservation, University of Montana, Missoula, MT USA; 18 IUCN Conservation Centre, Luc Hoffmann Institute, Gland, Switzerland

**Keywords:** Ecology, Environmental sciences, Careers

**To the Editor** — The COVID-19 pandemic poses major challenges for all sectors of society, including scientists faced with abrupt disruptions and redirections of research and higher education^1^. The consequences of this crisis will disproportionately impact early-career scientists; especially those from communities historically underrepresented, disadvantaged and/or discriminated in the fields of environmental sciences, including women, researchers from the Global South and persons with disabilities^2^. We call for a collective effort by the entire scientific community, especially those in leadership positions, to respond to the short- and long-term challenges of this crisis and to protect decades of efforts to build an inclusive scientific community^3^.

Diverse and inclusive scientific communities are more productive, innovative and impactful^4^, but also acutely threatened by the current crisis. Sudden increase in responsibilities for family care, teaching, supervision and administration particularly risks scientists from underrepresented groups becoming severely overburdened^5^. For example, women are more often responsible for service and student mentorship than their male colleagues in academia, resulting in increased workloads and fewer opportunities for career advancement^4,6^. The current crisis may also pose disproportionate existential threats to scientists about whose representation and equality is still too little known (for example, ethnic and racial minorities, LGBTQ+, and disabled individuals). Scientists with limited financial or technological resources who depend on temporary income or visas for their work are currently at a distinct disadvantage^7^, and need support to pursue educational and career opportunities.

**Fig. 1:**
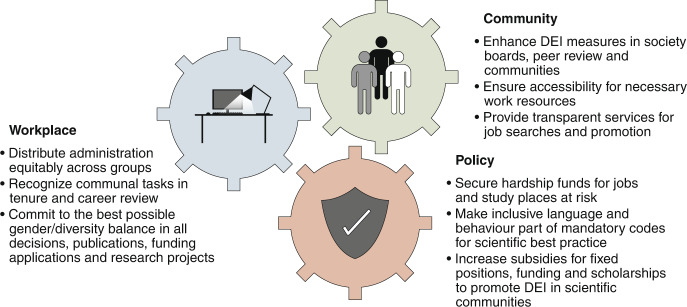
Actions are required at all science levels. Three ideas for the Academic Leadership to improve diversity, equity and inclusion (DEI) in the scientific workplace, at community and institute levels, as well as in broader policy and decision-making.

Inequalities based on racism and discrimination, such as the disturbing instances of racist attacks on people of Asian descent since the spread of the virus, will affect the international scientific community not only in the next weeks and months^8^, but over the long term. Coping with the current and long-term consequences of the pandemic requires courageous action at all levels of our scientific community (Fig. 1). The Academic Leadership is especially in demand for actively supporting and protecting the integrity of our field, and building a diversity, equity and inclusion focus into all COVID-19-related recovery efforts in scientific workplaces, communities and broader policies.

Fair distribution and recognition of communal tasks build the foundation for a supportive academic environment, but early-career scientists in precarious situations need more than that. Scientific policy and decision-makers need to set up support measures that protect inclusive scientific communities from economic recession, reduced job and funding availability, and increased competition. Increasing job security and resource accessibility creates more healthy work environments, intercepting emotional and financial stress caused by inequality^9,10^. Overcoming this pandemic requires a strong international scientific community that understands that diversity and equity are key factors in promoting healthy, resilient environments as the cornerstones of human health and well-being^9,11^.
